# Anatomical Proposal for Botulinum Neurotoxin Injection Targeting the Platysma Muscle for Treating Platysmal Band and Jawline Lifting: A Review

**DOI:** 10.3390/toxins14120868

**Published:** 2022-12-10

**Authors:** Kyu-Ho Yi, Ji-Hyun Lee, Kangwoo Lee, Hye-Won Hu, Hyung-Jin Lee, Hee-Jin Kim

**Affiliations:** 1 Wonju Public Health Center, Wonjusi 26417, Republic of Korea; 2 Division in Anatomy and Developmental Biology, Department of Oral Biology, Human Identification Research Institute, BK21 PLUS Project, Yonsei University College of Dentistry, 50-1 Yonsei-ro, Seoul 03722, Republic of Korea; 3 Department of Anatomy, Catholic Institute for Applied Anatomy, College of Medicine, The Catholic University of Korea, Seoul 06591, Republic of Korea

**Keywords:** botulinum neurotoxin, platysma band, platysma muscle, dermotoxin, injection point, face lifting

## Abstract

The platysma muscle is a thin superficial muscle that covers the entire neck and lower part of the face. The platysma muscle is the primary target muscle for botulinum neurotoxin injection therapy aimed at treating platysmal band and lower facial lifting. In the procedure of botulinum neurotoxin injection therapy, a lack of knowledge of the anatomy of the platysma muscle and the properties of botulinum neurotoxin can lead to side effects such as dysphagia, dysphonia, and weakness of the neck muscles. Anatomically safe injection sites have been proposed for the platysma muscle, and the appropriate injection technique has been reviewed. We proposed optimal injection sites based on the external anatomical features of the mandible. The aim of these proposal was to standardize the procedure for the effective use of botulinum neurotoxin injections by minimizing the dose unit and injection points and thereby preventing adverse events.

## 1. Introduction

**Figure 1:**
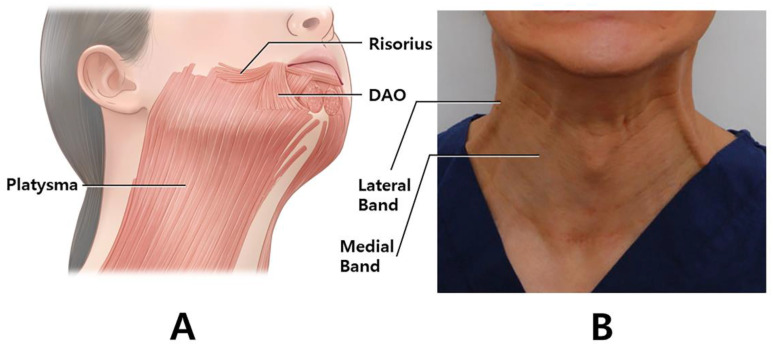
The image of the playsmal bands. The posterior and anterior platysmal bands are targeted for botulinum neurotoxin (**A**). Schematic image of the platysma muscle in oblique view. The muscle intermingles with the depressor anguli oris, depressor labii inferioris, mentalis, risorius, and orbicularis oris muscles (**B**). (DAO; depressor anguli oris).

*Clostridium botulinum* produces botulinum neurotoxin (BoNT), which prevents the release of acetylcholine at the motor end plate [1,2]. In aesthetic settings, BoNT is frequently used to target the platysma muscle to diminish the platysmal band or jawline lifting (Figure 1A). Platysma bands are two strips of muscle bands that are found in the hypercontraction status of the platysma muscle (Figure 1B). The bands get more prominent with age and become an aesthetic concern [3].

Jawline lifting is also an aesthetic treatment conducted with BoNT injection in the jawline, which is given in the platysma muscle [4].

The platysma muscle acts by pulling down the modiolus, corners of the mouth, and lower third of the face. Therefore, BoNT injection into the platysma muscle results in lifting of the lower third of the face and jawline contour, which improves the esthetic appearance by correcting the drooping corners of the mouth and jowls.

However, an overdose of BoNT may paralyze nearby muscles, causing dysphonia, dysphagia, and an asymmetrical appearance [5]. Inadvertent diffusion of BoNT into the depressor labii inferioris or risorius muscle may result in an asymmetric smile (Figure 1B). To prevent these undesirable results, anatomy-based precise injection in the platysma muscle and beginning the primary treatment on a lower quantity of BoNT are recommended. Recent studies on BoNT injection in specific locations for anatomical assessments of particular muscles have been conducted on the basis of external anatomical features [6,7].

Clinicians treating the platysma muscle need to have a good understanding of the anatomy of the jaw and neck. However, few anatomical findings have been reviewed with clinical application (location, injection technique, and dosage).

In this study, the authors reviewed the anatomical morphology of the platysma and the limited literature available to develop own approach to try to maximize benefit and minimize adverse effects of injecting BoNT into the platysma.

## 2. Anatomy of the Platysma Muscle

**Figure 2:**
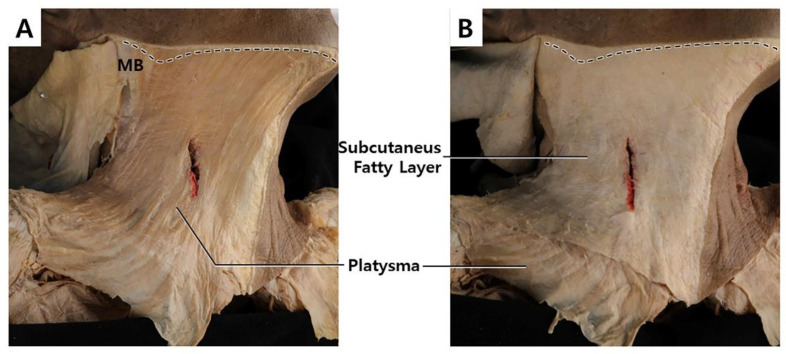
The dissected image of the platysma muscle in a cadaver (**A**) and revealing platysma muscle by removing subcutaneous tissue (**B**).

**Figure 3:**
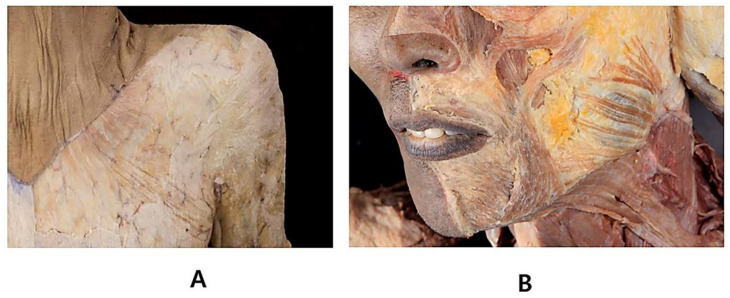
Platysma muscle continuously extends to the fascial structure of pectoralis major and anterior deltoid (**A**). The platysma also extends to mid-face region (**B**).

The platysma is a superficially located thin and broad muscle underneath the subcutaneous fatty layer, which covers most of the neck region (Figure 2A) [8]. The superior part of the platysma muscle, involved in expression, pulls the cheilion inferiorly and widens the lips. The inferior part of the platysma muscle acts by pulling the skin of the neck superiorly. Neural innervation is facilitated by the cervical branch of the facial nerve, and blood supply is facilitated by branches of the submental and suprascapular arteries. The platysma muscle originates from the fascial structure of the pectoralis major and ends at the anterior deltoid (Figure 3A). The muscle fibers superiorly blend medially into the depressor anguli oris, depressor labii inferioris, and lower lip, and laterally form the superficial musculoaponeurotic system (Figure 3B). 

In the middle of the neck, the platysma is absent in the lower region and partially exists in the upper region depending on the individual. The upper region of the middle neck has some partly decussated fibers, covering the submental region. A previous study revealed that 85% of medial platysmal fibers decussate in the submental region. In 43% of subjects, the decussating fibers extended more than 20 mm below the mandibular border. It was suggested that gobbler neck deformity can be present in individuals with no decussation or less decussation, along with sagging of nearby subcutaneous tissue. In an aesthetic setting, an anatomical structure called the superficial cervical fascia (investing layer) often refers to the subdermal layer (Figure 2B). The structure includes adipose tissue, lymph nodes, nerves, and vessels that are superficially located, including the platysma muscle. 

## 3. Platysmal Band Injection Points and Methods 

Two units (U) are injected per point, and five points are injected for each band, approximately 2 cm from each other along the muscular band. A total of 40 units are used on both sides of the medial and lateral bands. The type of the botulinum toxin proposed are type A, ona-botulinum toxins. The ona-botulinum toxin A was set as the gold standard since it was first approved and commercialized as BoNT. There are conversion rate to other dosing unit and dilution rate. [9] The author dilutes with 2.5 mL of normal saline to use at a concentration of 4 U/0.1 mL.

**Figure 4:**
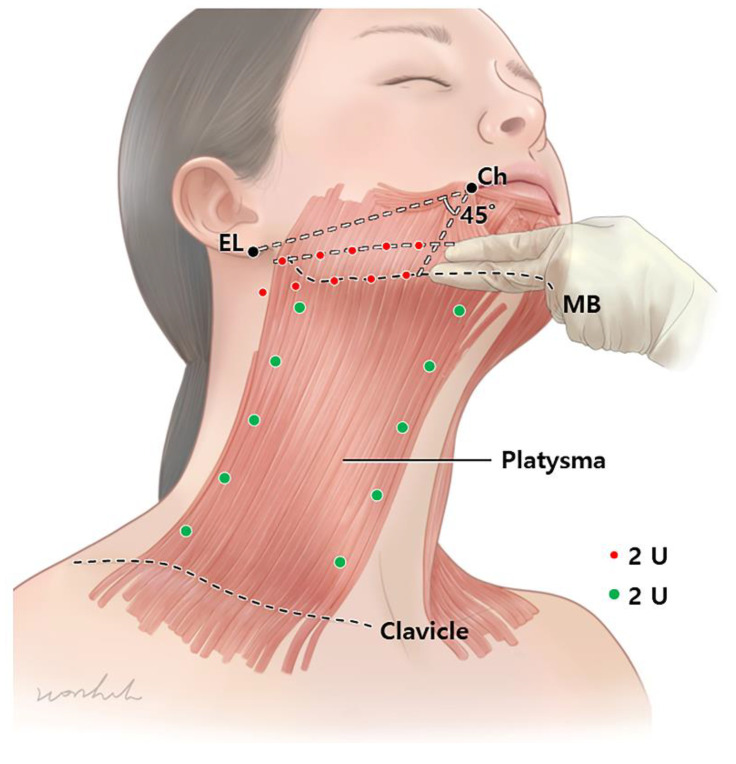
The jawline lifting, 2 U is injected per point. A total of 20 U of botulinum neurotoxin per side is injected. The injection should be in two lines (distance of two finger width), one right above the mandibular lower border and one below the line connecting the cheilion and ear lobule. For the platysmal bands, 2 U is injected per point (green dots). For injection, 5 points are placed for each band approximately 2 cm from each other along the muscular band. A total of 40 units are used for both sides of medial and lateral bands. (EL, ear lobe; Ch, Cheilion; MB, Mandibular border.).

Platysmal bands should be assessed primarily. First, patients are asked to make a gloomy face by pulling down hard on the mouth angle, such that the platysma muscle is hyperactivated. Then, the BoNT is directly injected into the muscular bands 5 points below the jawline to the clavicle. Following that, 2 U each is injected at 5 points along the medial and lateral bands (Figure 4). 

## 4. Jawline Lift (Nefertiti and “Microbotox”) Injection Points and Methods

For the jawline lifting, 2 U is injected per point. A total of 20 U of BoNT per side is injected. The injection should be in two lines, one right above the mandibular lower border and one below the line connecting the cheilion and ear lobule. The lines should have 5 points each, with a distance of 2 cm. To prevent diffusion into the depressor labii inferioris, injection into the mandibular lower border should be performed superior to the marionet line (45° from the cheilion). For the jawline lift, BoNT should be injected at the subdermal or intradermal levels. 

A total of 40 U is injected; however, in cases of severe drooping mouth and sagging jowl, an additional injection of the platysmal band would be helpful. 

In addition, a line outside 45° from the cheilion is recommended. Subsequently, 2 U is injected per point. A total of 20 U of BoNT per side is injected (Figure 4).

## 5. Discussion

The neck horizontal lines are innate lines that are present from birth at the anterior part of the neck, which cannot be treated with BoNT unlike the vertical platysmal band. However, the horizontal neck line can be managed by paralyzing the platysma muscle, which pulls the lower face and the subcutaneous tissue of the neck downward. The horizontal line of the neck is thought to be associated with retaining ligaments. Therefore, treating these retaining ligaments with subcision and filler injection may reduce the horizontal neck line [10,11,12]. 

Vertical platysmal bands are effectively treated with BoNT. Platysmal bands appear in two distinctive areas: anterior and posterior bands form at the medial and lateral borders of the muscle. Trevidic and Criollo-Lamilla conducted an observational study in 25 patients with unilateral facial palsy following otoneurosurgical treatment. They proved that patients with facial palsy did not have platysmal bands and that the vertical platysma bands are more related to hyperactivity of the muscle rather than relaxation of the platysma and skin laxity [13]. 

Injecting BoNT into the platysma muscle can improve not only the vertical bands, but also the drooping corners of the mouth and the jawline. The platysma muscle pulls down the modiolus, corners of the mouth, and lower face inferolaterally. Therefore, when the platysma muscle contracts, it causes sagging of the subcutaneous tissue, which causes a droopy mouth and jowls. Botulinum rebalancing, which adjusts the balance between elevators and depressors for the jawline, drooping corners of the mouth, and sagging jawline, leads to significant lifting by weakening the platysma muscle, which is a depressor. 

In 2007, Levy presented the concept of the “Nefertiti lift”, which involved injecting BoNT into the inferior border of the mandible [14,15]. In 2015, the “Microbotox” technique [15] was first described by Wu, which also targeted the inferior border of the mandible, with a shallow injection depth compared to that in the “Nefertiti lift”. Both deep intramuscular injection of the Nefertiti lift and superficial intradermal “Microbotox” injections showed satisfactory results in neck lifting and platysmal band treatment [16]. In a study comparing the effects of the two methods, Awaida et al. concluded that deep injection was appropriate in the platysmal band, whereas jawline lifting was effective when BoNT injected intradermally [17]. 

The injection range of the lower face region should depend on the extension of the platysma to the facial region. Shah et al. extended the platysma up to the mean of 4 cm superiorly from the inferior border of the mandible. [18] In a previous study, the platysma muscle has been extended up to the mid-face [19]. 

**Figure 5:**
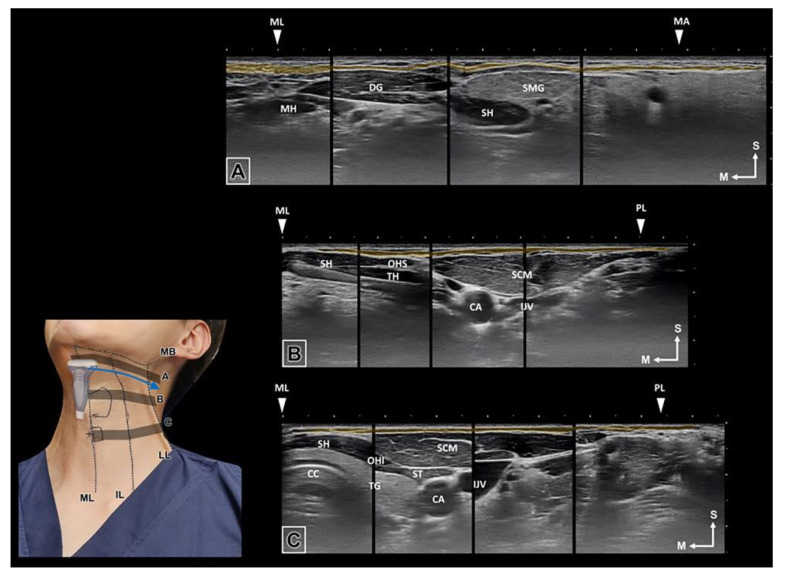
The ultrasonographic image of the platysma muscle was observed in blue arrow direction. The muscle lies from the midline to the vertical line from the mandibular angle and has been presented in the ultrasonographic image of three transverse planes: below the mandibular border (**A**), superior notch of the thyroid cartilage (**B**), and cricoid cartilage (**C**). (ML, midline; MH, Mylohyoid muscle; DG, Digastric muscle; SH, Sternohyoid muscle; SMG, Submandibular gland; MA, mandibular angle; CA, carotid artery; OHS, Omohyoid superior belly; OHI, Omohyoid inferior belly; IJV, Internal jugular vein; TH; Thyrohyiod muscle; TG; Thyroid gland; CC, Cricoid cartilage; IL, intermediate line; LL, lateral border of the lateral band).

Overdose of BoNT into the platysma muscle may cause dysphagia, dysphonia, and weakness of the neck muscles by diffusion of BoNT into the underlying muscles [20,21,22,23]. Furthermore, diffusion into nearby muscles, such as the depressor labii inferioris, may result in an asymmetric smile [24]. The ultrasonography in detecting the platysma muscle would be supportive option in injection guidance (Figure 5). 

Therefore, a lower dosage and more precise location of the injection should be proposed from an anatomical perspective. In addition, BoNT may become ineffective as a result of antibody production due to repeated BoNT injections [25,26,27,28]. In this review, the clinical aspect of platysma muscle anatomy has been dealt with, along with possible injection points and methods. The proposal suggests a minimal approach, and if the outcomes are unsatisfactory, an additional injection may be administered [21]. The limitation of the study was that there are insufficient clinical trials and literatures. The review was based on the anatomical information and no consensus panels to support a single approach to platysma injection, thereby, were not able to demonstrate the techniques we proposed were superior and safer to the other methods. Further, clinical study should be conducted. However, the study is significant in usage of minimal amount of BoNT and location are based on anatomical information that can be easily applied.

## 6. Conclusions

In conclusion, the review highlights the anatomical aspects of BoNT injections in the platysma muscle. For the platysma band, a total of 40 U for 20 points on both sides of the medial and lateral bands should be injected intramuscularly after palpation. For the jawline injection, a total of 40 U is injected at 20 points with subdermal injection, in two lines: above the lower border of the mandible, and the line connecting the cheilion and earlobe. 

## Data Availability

Not applicable.

## References

[1] Dessy L.A., Mazzocchi M., Rubino C., Mazzarello V., Spissu N., Scuderi N. (2007). An objective assessment of botulinum toxin A effect on superficial skin texture. Ann. Plast. Surg.

[2] Childers M.K. (2004). Targeting the neuromuscular junction in skeletal muscles. Am. J. Phys. Med. Rehabil..

[3] Sugrue C.M., Kelly J.L., McInerney N. (2019). Botulinum Toxin Treatment for Mild to Moderate Platysma Bands: A Systematic Review of Efficacy, Safety, and Injection Technique. Aesthetic Surg. J.

[4] Lorenc Z.P., Corduff N., van Loghem J., Yoelin S. (2022). Creating Lift in the Lower Face With Botulinum Toxin A Treatment: An Anatomical Overview With Videos and Case Studies Illustrating Patient Evaluation and Treatment. Aesthetic Surg. J. Open Forum.

[5] Witmanowski H., Blochowiak K. (2020). The whole truth about botulinum toxin—A review. Postepy Dermatol. Alergol.

[6] Yi K.H., Lee H.J., Seo K.K., Kim H.J. (2022). Botulinum neurotoxin injection guidelines regarding flap surgeries in breast reconstruction. J. Plast. Reconstr. Aesthetic Surg..

[7] Yi K.H., Lee J.H., Hu H.W., Kim H.J. (2022). Anatomical Proposal for Botulinum Neurotoxin Injection for Glabellar Frown Lines. Toxins.

[8] Baur D.A., Williams J., Alakaily X. (2014). The platysma myocutaneous flap. Oral Maxillofac Surg. Clin. North Am.

[9] Scaglione F. (2016). Conversion Ratio between Botox(R), Dysport(R), and Xeomin(R) in Clinical Practice. Toxins.

[10] Jacono A.A., Malone M.H. (2017). Characterization of the Cervical Retaining Ligaments During Subplatysmal Facelift Dissection and its Implications. Aesthetic Surg. J..

[11] Tseng F., Yu H. (2019). Treatment of Horizontal Neck Wrinkles with Hyaluronic Acid Filler: A Retrospective Case Series. Plast. Reconstr. Surg. Glob. Open.

[12] Lee H.-J., Ryu S.-Y., Ahn H.-J., Cho S.-W., Kim H.-J., Hu K.-S. (2017). Does Retaining Ligament Exist in the Neck. Korean J. Phys. Anthr..

[13] Trevidic P., Criollo-Lamilla G. (2017). Platysma Bands: Is a Change Needed in the Surgical Paradigm?. Plast. Reconstr. Surg..

[14] Levy P.M. (2007). The ‘Nefertiti lift’: A new technique for specific re-contouring of the jawline. J. Cosmet. Laser Ther..

[15] Wu W.T.L. (2015). Microbotox of the Lower Face and Neck: Evolution of a Personal Technique and Its Clinical Effects. Plast. Reconstr. Surg..

[16] Jabbour S.F., Kechichian E.G., Awaida C.J., Tomb R.R., Nasr M.W. (2017). Botulinum Toxin for Neck Rejuvenation: Assessing Efficacy and Redefining Patient Selection. Plast. Reconstr. Surg..

[17] Awaida C.J., Jabbour S.F., Rayess Y.A., El Khoury J.S., Kechichian E.G., Nasr M.W. (2018). Evaluation of the Microbotox Technique: An Algorithmic Approach for Lower Face and Neck Rejuvenation and a Crossover Clinical Trial. Plast. Reconstr. Surg..

[18] Shah A.R., Rosenberg D. (2009). Defining the facial extent of the platysma muscle: A review of 71 consecutive face-lifts. Arch. Facial. Plast. Surg..

[19] Bae J.H., Youn K.H., Hu K.S., Lee J.H., Tansatit T., Kim H.J. (2016). Clinical Implications of the Extension of Platysmal Fibers on the Middle and Lower Face. Plast. Reconstr. Surg..

[20] Holzer S.E., Ludlow C.L. (1996). The swallowing side effects of botulinum toxin type A injection in spasmodic dysphonia. Laryngoscope.

[21] Seo K.K. (2017). Botulinum Toxin for Asians.

[22] Brans J.W., de Boer I.P., Aramideh M., Ongerboer de Visser B.W., Speelman J.D. (1995). Botulinum toxin in cervical dystonia: Low dosage with electromyographic guidance. J. Neurol..

[23] Yiannakopoulou E. (2015). Serious and long-term adverse events associated with the therapeutic and cosmetic use of botulinum toxin. Pharmacology.

[24] Kassir M., Gupta M., Galadari H., Kroumpouzos G., Katsambas A., Lotti T., Vojvodic A., Grabbe S., Juchems E., Goldust M. (2020). Complications of botulinum toxin and fillers: A narrative review. J. Cosmet. Dermatol..

[25] Hsu T.S., Dover J.S., Arndt K.A. (2004). Effect of volume and concentration on the diffusion of botulinum exotoxin A. Arch. Dermatol.

[26] Kinnett D. (2004). Botulinum toxin A injections in children: Technique and dosing issues. Am. J. Phys. Med. Rehabil..

[27] Lepage D., Parratte B., Tatu L., Vuiller F., Monnier G. (2005). Extra- and intramuscular nerve supply of the muscles of the anterior antebrachial compartment: Applications for selective neurotomy and for botulinum toxin injection. Surg. Radiol. Anat..

[28] Pingel J., Nielsen M.S., Lauridsen T., Rix K., Bech M., Alkjaer T., Andersen I.T., Nielsen J.B., Feidenhansl R. (2017). Injection of high dose botulinum-toxin A leads to impaired skeletal muscle function and damage of the fibrilar and non-fibrilar structures. Sci. Rep..

